# Supporting Sustainable Food Consumption: Mental Contrasting with Implementation Intentions (MCII) Aligns Intentions and Behavior

**DOI:** 10.3389/fpsyg.2016.00607

**Published:** 2016-04-29

**Authors:** Laura S. Loy, Frank Wieber, Peter M. Gollwitzer, Gabriele Oettingen

**Affiliations:** ^1^Media Psychology Division, School of Communication, University of HohenheimStuttgart, Germany; ^2^Social Psychology and Motivation Division, Department of Psychology, University of KonstanzKonstanz, Germany; ^3^Centre for Health Sciences, School of Health Professions, Zurich University of Applied SciencesWinterthur, Switzerland; ^4^Motivation Lab, Psychology Department, New York UniversityNew York, NY, USA; ^5^Educational Psychology and Motivation Division, Department of Psychology, University of HamburgHamburg, Germany

**Keywords:** sustainable consumption, meat consumption, intention-behavior gap, behavior change intervention, mental contrasting, implementation intention

## Abstract

With growing awareness that sustainable consumption is important for quality of life on earth, many individuals intend to act more sustainably. In this regard, interest in reducing meat consumption is on the rise. However, people often do not translate intentions into actual behavior change. To address this intention-behavior gap, we tested the self-regulation strategy of mental contrasting with implementation intentions (MCII). Here, people identify and imagine a desired future and current obstacles standing in its way. They address the obstacles with if-then plans specifying when, where, and how to act differently. In a 5-week randomized controlled experimental study, we compared an information + MCII intervention with an information-only control intervention. As hypothesized, only MCII participants’ intention of reducing their meat consumption was predictive of their actual reduction, while no correspondence between intention and behavior change was found for control participants. Participants with a moderate to strong intention to reduce their meat consumption reduced it more in the MCII than in the control condition. Thus, MCII helped to narrow the intention-behavior gap and supported behavior change for those holding moderate and strong respective intentions.

## Introduction

Sustainable consumer behavior aims to enable a good present and future quality of life on earth through a wise use of resources (Jackson, 2005; UNDESA, 2010; UNEP, 2012). Among dietary choices, consuming less meat qualifies as sustainable behavior change, because plant-based products require less land, fossil fuel, and water resources. They cause lower pollution as well as greenhouse gas emissions than meat products (Jungbluth et al., 2000; Leitzmann, 2003; Pimentel and Pimentel, 2003; FAO, 2006; Goodland and Anhang, 2009; Popp et al., 2010; UNEP, 2010; IPCC, 2014; Tilman and Clark, 2014; Westhoek et al., 2014).

For a growing number of individuals, reducing meat intake has become a means to consume more sustainably (Kalof et al., 1999; White et al., 1999; Fox and Ward, 2008; Vinnari and Tapio, 2009). Next to environmental protection, other reasons speak for and are named by individuals for reducing meat consumption as, for example, animal-ethical aspects (Pluhar, 2010), health aspects (ADA, 2009), or political and social aspects such as global nutrition security (Leitzmann, 2003; UNEP, 2010; for an overview see Ruby, 2012). However, despite the rising interest in reducing meat intake (Povey et al., 2001; Grunert, 2006; Vermeir and Verbeke, 2006; Cordts et al., 2013), many people experience great difficulties preventing them to change their nutritional routines (Lea and Worsley, 2001; Lea et al., 2006).

Thus, the question arises what kind of intervention could support individuals to overcome these personal difficulties in order to reduce their meat intake. Many theoretical models of behavior change and derived interventions assume *intentions* (i.e., “self-instructions to perform particular behaviors or to obtain certain outcomes”; Webb and Sheeran, 2006, p. 249) as immediate predictors of behavior (Fishbein and Ajzen, 2010). However, although forming the intention “I want to reduce my meat consumption!” is a necessary first step to behavior change, it may not suffice. Several mechanisms can interfere between intention formation and behavior change, as for example following established habits, giving in to temptations, missing good opportunities for action, or simply forgetting about intentions (Gollwitzer and Sheeran, 2006; Ji and Wood, 2007; Wood and Neal, 2009). Getting started to reduce meat intake can, for example, be hindered by a lack of knowledge on plant-based alternatives or by regularly cooking with other people who insist on having meat. Staying on track to eat differently can be threatened by giving in to temptations such as seeing a delicious meat dish on the menu of a restaurant. These mechanisms result in the so-called *intention-behavior gap* (Sheeran, 2002): formulated intentions do not correspond to action; the strength of agreement with the statement “I want to change my behavior” is not or weakly correlated with the degree of subsequent behavior change.

The intention-behavior gap is illustrated by a meta-analysis on the determinants of pro-environmental behavior (Bamberg and Möser, 2007), in which intentions to perform a behavior accounted only for 27% of variance in actual behavior. Moreover, this gap has also been found for interventions aiming to change participants’ intentions. In a meta-analysis of experimental studies (Webb and Sheeran, 2006), a medium-to-large change in intention resulted only in a small-to-medium change in behavior.

The present study suggests a self-regulation approach, focusing on how individuals can effectively guide their behavior toward goal attainment (e.g., Karoly, 1993). One strategy to narrow the intention-behavior gap consists in forming *implementation intentions* (IIs; Gollwitzer, 1993, 1999, 2014). Here, individuals make if-then plans specifying when, where, and how to act (“If I encounter situation X, then I will perform behavior Y”) in order to implement a certain intention (“I intend to achieve goal Z”).

As a consequence of forming IIs, individuals should easily recognize the anticipated situation when it arises and then initiate the linked action immediately, efficiently, and without requiring a conscious intent to act in the critical moment (e.g., II effects are still evident when the critical cue is presented subliminally or when the respective goal is activated outside of awareness; Sheeran et al., 2005; Bayer et al., 2009). In line with these assumptions, studies confirmed that the mental representation of the anticipated situation becomes highly accessible (Aarts et al., 1999; Wieber and Sassenberg, 2006; Webb and Sheeran, 2007; Achtziger et al., 2012) and the link between situation and action is strengthened (Brandstätter et al., 2001; Webb and Sheeran, 2007, 2008; Bayer et al., 2009; Mendoza et al., 2010). These cognitive consequences of forming IIs are rather stable over time (Papies et al., 2009).

Meta-analyses showed that IIs have medium to large effects on goal attainment (Gollwitzer and Sheeran, 2006; Adriaanse et al., 2010b; Bélanger-Gravel et al., 2013; Chen et al., 2015). They help overcome typical problems of goal striving such as getting started with the intended behavior as well as staying on track when encountering obstacles (for summaries see Gollwitzer and Sheeran, 2009; Gollwitzer et al., 2010a,b; Gollwitzer and Oettingen, 2011).

Regarding consumption-related intentions, IIs have supported individuals to drink less alcohol (e.g., Armitage, 2009; Chatzisarantis and Hagger, 2010; Armitage and Arden, 2012; Hagger et al., 2012), quit and prevent smoking (e.g., Armitage, 2007b; Conner and Higgins, 2010), or eat healthier (e.g., De Nooijer et al., 2006; Armitage, 2007a; Knäuper et al., 2011; Chapman and Armitage, 2012). Interestingly, II interventions to reduce consumption of certain foods seem to benefit from personalization. Adriaanse et al. (2009) found that IIs specifying personally relevant critical cues for unwanted snacking in the if-part reduced unhealthy snacking behavior (Study 2), while IIs specifying experimenter-defined cues in the if-part did not (Study 1).

One way to support individuals’ efforts to find and select personally relevant II cues is to precede IIs with *mental contrasting* (MC; Oettingen, 2000, 2012; Oettingen et al., 2001). Here, individuals identify a personal wish or goal (e.g., holding one vegetarian day a week), identify and imagine the most positive future outcome of goal attainment (e.g., contributing to a healthy environment), and identify the main personal obstacle currently impeding their goal attainment (e.g., not knowing any meat-free recipes).

Mental contrasting is shown to affect behavior by changing non-conscious cognitive and motivational processes (summary by Oettingen, 2012). In line with this assumption, MC was observed to increase the strength of individuals’ implicit mental association between the wished-for future or goal and the obstacle as well as between the obstacle and the instrumental means to overcome the obstacle, given that chances of reaching the future are high. These mental associations in turn predicted goal pursuit (Kappes et al., 2012; Kappes and Oettingen, 2014). In addition, individuals with high chances of realizing the future who performed MC (versus relevant control groups) implicitly categorized their present reality as an obstacle and more readily detected an obstacle toward goal attainment as such (Kappes et al., 2013). Moreover, MC affected implicit and explicit indicators of motivational processes. For example, when chances of success were high, MC increased individuals’ implicit (systolic blood pressure) and explicit (self-reported) energization, which in turn supported the desired behavior changes (e.g., stress coping; Oettingen et al., 2009a; Sevincer et al., 2014).

Mental contrasting has helped individuals to reach a variety of wishes and goals including success in academic performance (Gollwitzer et al., 2011), finding integrative solutions in a negotiation task (Kirk et al., 2011), helping behavior (Oettingen et al., 2010), and exercising (Johannessen et al., 2011; Sheeran et al., 2013). Regarding consumption-related goals, MC has supported people to eat fewer calories (Johannessen et al., 2011), initiate a reduction of cigarette consumption (Oettingen et al., 2009b), and manage dieting behavior of individuals with Type II diabetes (Adriaanse et al., 2013; summaries by Oettingen, 2012; Oettingen and Schwörer, 2013).

Complementing MC instructions with II instructions results in the intervention strategy of mental contrasting with implementation intentions (MCII; overviews by Oettingen, 2012, 2014; Oettingen et al., 2013). This combination appears especially well suited to support behavior change, because MC helps to identify important personal obstacles for behavior change that can then be addressed by IIs. Individuals can link an obstacle as situational cue in the if-part to specific actions to overcome the obstacle in the then-part. MC *per se* implicitly connects the obstacle to an effective instrumental response so that people can master the obstacle more easily (Kappes et al., 2012). However, adding an explicitly formulated plan of how to overcome the obstacle in the form of an II additionally benefits their goal pursuit and helps to translate intentions into actual behavior change.

Mental contrasting with implementation intentions has been applied to facilitate behavior change in several domains including exercise (Stadler et al., 2009), physical capacity of chronic back pain patients (Christiansen et al., 2010), and the promotion of healthy consumption in terms of eating more fruits and vegetables (Stadler et al., 2010), and snacking less (Adriaanse et al., 2010a). In the latter study, female participants were randomly assigned to one of three intervention conditions: MC, II, or MCII. In support of the effectiveness of combining MC and II, MCII participants reported being more successful at diminishing their unhealthy snacking 1 week after the intervention than participants who engaged in MC or II alone. Individuals in both conditions involving MC experienced more clarity about the critical obstacles for a healthier diet. This clarity in turn was related to success in reducing their habit (Adriaanse et al., 2010a, Study 2).

The present research examines whether MCII can support the translation of individuals’ intentions to consume less meat into actual behavior. We think of MCII as a well-suited technique to narrow individuals’ intention-behavior gap. In order to test this assumption, we conducted a 5-week longitudinal randomized controlled experimental study, in which participants reported their daily meat consumption in food diaries. We compared the effectiveness of two different intervention formats: (1) the classical information intervention in an information-only control condition (see e.g., Luszczynska et al., 2007; Stadler et al., 2010), and (2) the self-regulation intervention MCII in an information + MCII condition. We hypothesized that intentions predict behavior change better in the MCII condition than the control condition; in other words, the intervention condition (control vs. MCII) should moderate the intention-behavior relation.

## Materials and Methods

### Participants

The study was advertised on the campus of a German university as a study on meat consumption. In the announcement, we indicated that we looked for participants who were no vegetarians and were proficient in the German language. To address our research question, we draw on the data of 60 members of the university (*n* = 45 female) with a mean age of 22.3 years (*SD* = 4.25; range 18–49 years).^1^ Most of them were students of various majors (psychology major *n* = 29). They received either financial compensation or class credit.

### Study Design

We compared the effectiveness of two interventions in supporting an intended reduction in meat consumption: an information-only control intervention and an information + MCII intervention. Participants filled in diaries on their meat consumption in the week before the intervention (Baseline Diary), the week after the intervention (Follow-up 1 Diary), and the 4th week after the intervention (Follow-up 2 Diary). The differences in meat consumption served as dependent variables. Furthermore, we analyzed the strength of intention to reduce meat consumption at baseline as predictor of behavior change and intervention condition (control vs. MCII) as a moderator of the intention-behavior relation.

### Procedure

The university’s ethics committee approved the study. We used a fixed randomization to assign participants to the experimental conditions. To enhance standardization, we held all meetings with participants in the same laboratory and used written instructions. Each participant followed a study schedule of 5 weeks including two laboratory meetings.

#### Baseline Measurement

Experimenter 1 conducted the first laboratory meetings, during which she was left unaware of condition. She informed participants about the study procedure and let them sign the consent form. Furthermore, participants answered Baseline-Questionnaire 1 containing variables we used to characterize the sample and to conduct randomization checks. Next to demographic variables, we asked participants to estimate their *past average meat consumption* (see Berndsen and Van der Pligt, 2005). Participants indicated how many days a week they usually eat meat at breakfast, lunch, dinner, and as snacks, and then specified the average weight of meat consumed for each meal (none, less than 50 g, 50–100 g, 100–150 g, 150–200 g, 200–250 g, 250–300 g, and more than 300 g). Our application of the *Self-Report Index of Habit Strength* (Verplanken and Orbell, 2003) contained 12 items. One example was: Eating meat is something I do without thinking; 1 (*doesn’t apply at all)* to 7 (*fully applies*). Cronbach’s α was 0.91 and we computed an average score for each person.^2^

Before leaving, participants received a paper-based 7-day diary on their meat consumption. They started to use it right after the first meeting in Week 1 of the study schedule (Baseline Diary; see Bolger et al., 2003, for an overview on diary methods). Seven-day periods are recommended for measuring food consumption, because of possible systematic variations over the course of a week (De Castro, 1994; Wolper et al., 1995). Adapting an approach from Berndsen and Van der Pligt (2005) for measuring meat intake, each day included a column for breakfast, lunch, dinner, and snacks (hence, each 1-week diary contained 28 items). We asked participants to indicate for each of these categories the weight of meat consumed (none, less than 50 g, 50–100 g, 100–150 g, 150–200 g, 200–250 g, 250–300 g, and more than 300 g). Examples of seven typical dishes and the respective weight were given as orientation (e.g., sausage about 100–120 g). We additionally encouraged participants to consult the weight information on packaging. Using the midpoint of the weight categories (e.g., 75 g for the category 50–100 g), we calculated a score for the average amount of meat consumed per day.

One week later, three different experimenters, who were unaware of content and hypotheses of the study, had the second laboratory meetings with the participants. Participants answered Baseline-Questionnaire 2, which contained a measure of the strength of intention to reduce meat consumption (i.e., the assumed predictor of behavior change). The measure contained four items following recommendations by Fishbein and Ajzen (2010): I intend to eat less meat in the coming weeks; I would like to reduce my meat consumption in the coming weeks; I will eat less meat in the coming weeks; and I will try to consume less meat in the coming weeks; 1 (*doesn’t apply at all*) to 7 (*fully applies*). Cronbach’s α was 0.98 and we computed an average score for each person.

#### Interventions

We provided the interventions during the second meeting. All participants read a text providing information about meat consumption. The information text was drawing on an article and a book written by a renowned German nutritionist (Leitzmann and Keller, 2010; Leitzmann, 2011). It not only addressed environmental consequences of meat consumption but also other arguments for reduced consumption that have been identified in research on individuals’ reasons for eating less meat (e.g., Leitzmann and Keller, 2010; Ruby, 2012). These further arguments referred to ethical, health, and social/political aspects of meat consumption including references to scientific publications.^3^

In the MCII condition, written instructions additionally led them through the MCII procedure. The MCII material was constructed similar to standard MCII interventions (Stadler et al., 2009, 2010; Oettingen et al., 2013). We explained the steps of MC with examples and then asked participants to write down their own personal thoughts regarding each step. In Step 1, we asked participants to determine a personal goal regarding their meat consumption (e.g., halving meat consumption or holding one vegetarian day a week). In Step 2, they had to state positive outcomes they related to attaining this goal (e.g., reducing environmental impact), then identify the best outcome, and imagine events and experiences associated with this best outcome. In Step 3, we asked them to identify and write down obstacles of present reality hindering goal achievement (e.g., habitual meat consumption in specific situations, lack of knowledge on alternatives), then identify their two main obstacles, and imagine events and experiences associated with these two main obstacles. Finally in Step 4, we suggested the formulation of IIs following Stadler et al. (2009, 2010). Two types of IIs were explained with examples. In the *strategy to overcome the obstacle*, participants should consider IIs specifying a behavior to overcome the two main obstacles which they had identified during MC in Step 3 (e.g., If I come home after sports with an appetite for meat, then I will cook with only half the amount of meat but more vegetables). In the *strategy to prevent the obstacle* from occurring, we suggested to explicate a situation to prevent the obstacle in the if-part, followed by a respective response in the then-part (e.g., If I write down my shopping list before going to the supermarket on Saturday, then I will look up a vegetarian recipe). We asked participants to formulate four personal IIs: for each of their two main obstacles they should write down one obstacle overcoming II and one obstacle preventing II. Thus, in sum, individuals wrote down (1) a personal goal, (2) positive outcomes of goal achievement, the best outcome, and respective associations, (3) obstacles of goal achievement, their two main obstacles, and respective associations, and finally (4) four IIs addressing their two main obstacles (see Supplementary Material for the exact wording).

#### Dependent Variables

At the end of the second meeting, participants received two further 7-day diaries identical to the Baseline Diary, one for Week 2 starting on the day after the intervention (Follow-up 1 Diary), one for Week 5 starting 3 weeks after the intervention (Follow-up 2 Diary). Altogether, we computed three meat consumption scores in g/day (Baseline Diary, Follow-up 1 Diary, and Follow-up 2 Diary) as well as two meat reduction scores in g/day (Meat Reduction Score 1: Baseline Diary – Follow-up 1 Diary; Meat Reduction Score 2: Baseline Diary – Follow-up 2 Diary). In Weeks 3 and 4, participants did not fill in any diaries. We asked participants to drop their diaries anonymously in a provided mailbox.

#### Additional Variables

Finally, participants received a link to an online questionnaire containing additional variables at the end of Week 5. This questionnaire served to rule out alternative explanations that differences between conditions regarding participants’ experiences of the study rather than the MCII intervention caused possible differences in meat consumption. We asked participants the following additional items: How sincerely did you answer the questions in the study? 1 (*not at all*) to 7 (*very much*), and Participating in the study was interesting for me! 1 (*doesn’t apply at all)* to 7 (*fully applies)*. Moreover, we assessed perceived experimenter demand to reduce meat consumption (see Adriaanse et al., 2010a): How much did the research staff conducting the study want you to reduce your meat consumption? 1 (*not at all*) to 7 (*very much*). In order to assess participants’ reasons behind reducing meat consumption, we additionally included the following four questions: How important do you find health aspects/animal-ethical aspects/environmental aspects/and social and political aspects of meat reduction? 1 (*not at all*) to 7 (*very much*).

## Results

### Participant Flow and Missing Values

All 60 participants handed in their Baseline Diary after Week 1 and returned to the second meeting that included the intervention. Then, *n* = 58 handed in their Follow-up 1 Diary covering Week 2, and *n* = 55 their Follow-up 2 Diary covering Week 5; *n* = 56 answered the final online questionnaire at the end of Week 5. For each follow-up diary as well as the online questionnaire, we performed a 2 (Attrition: response vs. non-response) × 2 (Condition: control vs. MCII) contingency table chi-square test, which revealed no differential attrition for the conditions, χ^2^s(1) ≤ 2.07, *p*s ≥ 0.492. A missing value analysis showed that item non-response was below 5% for all variables; Little’s Missing Completely At Random (MCAR) Test was not significant. It can therefore be assumed that missing variables occurred at random (Little and Rubin, 2002). For the sake of statistical power and in order not to exclude individuals due to few missing items, we applied maximum likelihood estimation. We generated a second data set including expectation maximization (EM) estimated values for all item non-response, but not wave non-response data (see Schafer and Graham, 2002). Results are reported for this data set and the *n* = 55 participants completing the study.

### Data Analyses

#### Descriptives and Randomization Check

Before the intervention, participants reported on average a moderate habit strength (*M* = 3.96, *SD* = 1.20, range 1.50–6.14) and a moderate intention to reduce meat consumption (*M* = 4.05, *SD* = 1.80, range 1–7). More specifically, our study comprised participants whose strength of intention was moderate to strong (i.e., 61.7% of participants reported a strength of intention ≥ 4.00 on our scale ranging from 1 to 7) as well as participants whose strength of intention was rather weak (i.e., 38.3% < 4.00). Reported past consumption was *M* = 110 g/day (*SD* = 60.1, range 14–257); consumption as assessed by the Baseline Diary was *M* = 97 g/day (*SD* = 47.7, range 11–207). All *t*-tests used to compare the conditions on these baseline variables as well as age were non-significant, *t*s(53) ≤ 1.72, *p*s ≥ 0.091. A chi-square test for gender did not reveal any difference between conditions either, χ^2^(1) = 0.34, *p* = 0.746. Hence, we can assume a successful randomization. Average meat reduction scores in our sample were *M* = 36 g/day (*SD* = 39.8, range -57 to -139) at Follow-up 1 and *M* = 38 g/day (*SD* = 40.9, range -32 to 161) at Follow-up 2. **Table 1** displays the average intention to reduce meat consumption as well as meat consumption levels at baseline, Follow-up 1, and Follow-up 2, differentiated for the interventions and including meat reduction scores.

**Table 1:** Means and standard deviations for the intention to reduce meat consumption, meat consumption levels, and meat reduction in g/day.

Variable		Control	MCII	*t*
Baseline intention	*M(SD)*	3.81 (1.97)	4.29 (1.59)	0.98
Baseline consumption	*M(SD)*	86.7 g (45.4)	108.5 g (48.3)	1.72
Follow-up 1 consumption	*M(SD)*	60.3 g (39.7)	63.2 g (34.9)	0.29
Follow-up 1 reduction	Δ*M(SD)*	26.4 g (38.2)	45.2 g (39.1)	1.79
Follow-up 2 consumption	*M(SD)*	59.7 g (42.4)	58.2 g (38.2)	-0.14
Follow-up 2 reduction	Δ*M(SD)*	27.0 g (39.4)	50.3 g (39.6)	2.19
	*n*	28	27	

*MCII, mental contrasting with implementation intentions.*

#### Reduction of Meat Consumption

Participants in the control condition reduced meat consumption at Follow-up 1 (i.e., in Week 2), *t*(27) = 3.66, *p* = 0.001, *d* = 0.63, as well as Follow-up 2 (i.e., in Week 5), *t*(27) = 3.62, *p* = 0.001, *d* = 0.63. So did participants in the MCII condition at Follow-up 1, *t*(26) = 5.90, *p* < 0.001, *d* = 1.09, as well as Follow-up 2, *t*(26) = 6.60, *p* < 0.001, *d* = 1.18. Whereas MCII participants did not have a significantly higher reduction of their meat consumption than the control participants at Follow-up 1, *t*(53) = 1.79, *p* = 0.079, *d* = 0.49, they did so at Follow-up 2, *t*(53) = 2.19, *p* = 0.033, *d* = 0.61. However, it has to be noted that the higher reduction in the MCII group did not result in lower consumption levels in an absolute sense in the follow-up measures, as the meat consumption scores in the MCII group were comparatively higher at baseline (although not statistically significant).

#### MCII’s Impact on the Intention-Behavior Relation

Next, we tested our prediction that the intention-behavior consistency differs between the control and the MCII intervention. In a first step, we determined the correlation between participants’ intention of reducing meat consumption and their actual meat reduction. For information + MCII participants, intention correlated with behavior change at Follow-up 1 (*r* = 0.54, *p* = 0.003) and Follow-up 2 (*r* = 0.53, *p* = 0.004). In contrast, for information-only control participants, intention correlated with behavior change neither at Follow-up 1 (*r* = -0.06) nor at Follow-up 2 (*r* = 0.14, *p*s ≥ 0.490).

In a second step, we conducted two moderated linear regression analyses using the SPSS-Macro PROCESS by Hayes (2012). We regressed meat reduction at Follow-up 1 as well as Follow-up 2 on condition (control vs. MCII), intention, and the Condition × Intention interaction term. Condition and intention were mean centered (see Aiken and West, 1991; Hayes, 2013), and 95% confidence intervals (CI) for the estimates were computed through 1,000 bootstrapped samples. We report unstandardized beta coefficients as recommended by Whisman and McClelland (2005).

**FIGURE 1:**
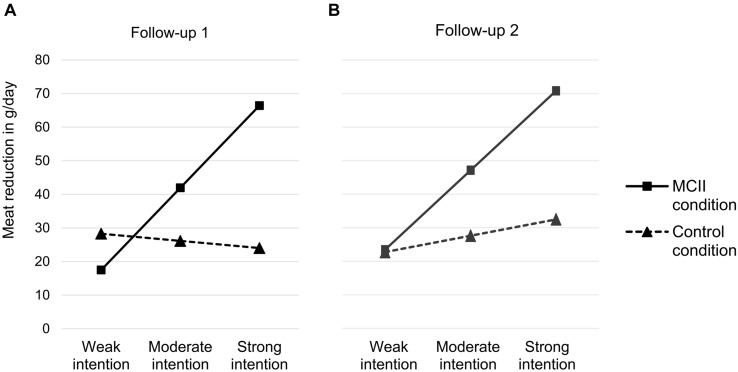
**Simple slopes for intention to reduce meat consumption differentiated for intervention condition (control vs. MCII) in the 1st week (A) and 4th week (B) after the intervention.** Meat reduction values of this display were calculated for weak intention (*M* - 1 *SD*), moderate intention (*M*) and strong intention (*M* + 1 *SD*). MCII, mental contrasting with implementation intentions.

At Follow-up 1, the model explained 20.1% of variance in meat reduction, *F*(3,51) = 4.83, *p* = 0.005 (see **Table 2**). We observed the predicted Condition × Intention interaction effect, *p* = 0.006. In the control condition, intention did not predict the reduction in meat consumption, *p* = 0.745, but it did in the MCII condition, *B* = 13.62, *SE* = 3.71, 95% CI [6.16,21.08], *t*(53) = 3.67, *p* < 0.001 (see **Figure 1**). To determine the intention level necessary for an additional effect of the MCII intervention on meat reduction, we reversed our analysis with condition as predictor and intention as moderator. We applied the Johnson-Neyman technique to determine the value along the continuum of the moderator intention at which the effect of the predictor condition transitions from statistically non-significant to significant (Hayes and Matthes, 2009; Hayes, 2013). We found that the effect of the MCII-intervention transitioned from statistically non-significant to significant at a intention level of 4.60 on our scale ranging from 1 to 7, *B* = 24.03, *t*(53) = 2.26, *p* = 0.028.

**Table 2:** Linear regression of meat reduction on condition (Control vs. MCII), intention, and the interaction term.

Variable	β	*SE*	*t*	*p*	LLCI	ULCI
**Follow-up 1**						
Condition (Control vs. MCII)	15.82	9.90	1.57	0.116	-3.06	35.69
Intention	6.08^∗^	2.59	2.34	0.023	0.78	11.10
Condition × Intention	14.81^∗∗^	5.19	2.85	0.006	4.39	25.23
**Follow-up 2**						
Condition (Control vs. MCII)	19.50	10.20	1.91	0.062	-0.99	39.99
Intention	7.86^∗∗^	2.52	3.12	0.003	2.80	12.92
Condition × Intention	10.48^∗^	5.06	2.07	0.044	0.32	20.64

*MCII, mental contrasting with implementation intentions. Betas are unstandardized coefficients. Condition was coded 0 = Control, 1 = MCII. Condition and Intention were then mean centered. LLCI, lower level 95% confidence interval, ULCI, upper level 95% confidence interval. ^∗^p < 0.05, ^∗∗^p < 0.01.*

Running the same analyses for Follow-up 2, the model explained 21.9% of variance in meat reduction, *F*(3,51) = 4.69, *p* = 0.006 (see **Table 2**). Again, we found the hypothesized Condition × Intention interaction effect, *p* = 0.044. In the control condition, intention did not predict the reduction in meat consumption, *p* = 0.403, but it did in the MCII condition, *B* = 13.20, *SE* = 3.90, 95% CI [5.37,21.03], *t*(53) = 3.38, *p* = 0.001 (see **Figure 1**). The additional effect of the MCII-intervention on meat reduction transitioned from statistically non-significant to significant at an intention level of 4.27, *B* = 22.17, *t*(53) = 2.03, *p* = 0.048. In line with our hypothesis, MCII supported the translation of intentions into behavior at Follow-up 1 and Follow-up 2. Individuals with a moderate to strong intention to reduce their meat consumption reduced it more in the MCII condition than the control condition.

Even though we successfully randomized participants to conditions and baseline meat consumption did not significantly differ between both groups, the descriptive difference between the two groups might still appear relevant. To test the stability of our pattern of results, we thus ran a matching analysis regarding baseline meat consumption (Van Casteren and Davis, 2007). This analysis resulted in two smaller datasets (*n* = 23 per condition) with *M* = 96.04 (*SD* = 44.38) in the control condition and *M* = 98.04 (*SD* = 43.54) in the MCII condition. We repeated our moderated regression analyses for these matched subgroups. For Follow-up 1, we observed the Condition × Intention interaction effect, *p* = 0.024. In the control condition, intention did not predict the reduction in meat consumption (*p* = 0.716), but it did in the MCII condition, *B* = 11.70, *SE* = 3.63, 95% CI [4.38,19.02], *t*(44) = 3.23, *p* = 0.002. For Follow-up 2, the Condition × Intention interaction effect did not reach significance, *p* = 0.307. Still, the pattern remained equivalent: in the control condition, intention did not predict the reduction in meat consumption (*p* = 0.431), but it did in the MCII condition, *B* = 9.45, *SE* = 3.05, 95% CI [3.30,15.60], *t*(44) = 3.10, *p* = 0.004.

#### Additional Variables

In order to rule out the alternative explanation that participants in the two conditions had experienced the study differently, we compared participants’ answers in the final online-questionnaire (*n* = 54, see **Table 3**). There were no differences between the MCII and control condition in perceived experimenter demand by research staff members, whether participating was perceived as interesting, and sincerity of participating, *t*s(52) ≤ 1.82, *p*s ≥ 0.075. Also with regards to reasons for reducing meat consumption, there were no differences in the importance participants attributed to the respective aspects between conditions, *t*s(52) ≤ 1.05, *p*s ≥ 0.300. Together, these findings suggest that the additionally assessed variables cannot account for the observed differences in the reduction in meat consumption between conditions. Interestingly, environmental aspects of reducing meat consumption (*M* = 5.74, *SD* = 1.36) received a higher rating of importance compared to social and political (*M* = 5.13, *SD* = 1.74), animal-ethical (*M* = 4.69, *SD* = 1.80), and health aspects (*M* = 4.24, *SD* = 1.69), *t*s(53) ≥ 3.03, *p*s ≤ 0.004.

**Table 3:** Means and standard deviations of additional variables in the control and MCII condition.

Variable		Control	MCII	*t*
Demand by research staff	*M(SD)*	4.22 (1.63)	3.78 (1.85)	0.94
Interestingness of participation	*M(SD)*	5.85 (1.17)	5.78 (1.28)	0.22
Sincerity of participation	*M(SD)*	6.59 (0.75)	6.22 (0.75)	1.82
Importance environmental aspects	*M(SD)*	5.78 (1.48)	5.70 (1.27)	0.20
Importance social/political aspects	*M(SD)*	5.07 (1.86)	5.19 (1.64)	-0.23
Importance animal-ethical aspects	*M(SD)*	4.48 (1.95)	4.89 (1.65)	-0.83
Importance health aspects	*M(SD)*	4.48 (1.78)	4.00 (1.59)	1.05
	*n*	27	27	

*MCII, mental contrasting with implementation intentions.*

## Discussion

We observed that the self-regulation intervention strategy of MCII (Oettingen, 2012; Oettingen et al., 2013) translated participants’ intentions to reduce meat intake into actual behavior change. In line with our hypothesis, participants’ intentions of reducing their meat consumption in the MCII condition were more predictive of their actual reduction than those in the information only control condition. This result supports our hypothesis that MCII narrows the intention-behavior gap. Participants supported with MCII reduced their meat consumption in correspondence with their prior intentions already in the 1st week after the intervention and sustained the intention-consistent consumption level 4 weeks later. By applying MCII to sustainable eating behavior in the form of reducing meat intake, our study extends prior research on the MCII intervention in other domains such as studying (Duckworth et al., 2011, 2013; Gawrilow et al., 2013), exercising (Christiansen et al., 2010), relating to one’s partner (Houssais et al., 2013), or negotiating the sale of a car (Kirk et al., 2013).

### MCII Puts Behavior in Line with Intentions

In addition, our study shows that the responsiveness to the strength of the goal intentions reported for the II technique (i.e., only strong goal intentions benefit from making if-then plans; Sheeran et al., 2005) is also valid for MCII as a combined intervention. As our study comprised participants whose strength of intention was moderate to strong (i.e., 61.7% of participants reported a strength of intention ≥ 4.00 on our scale ranging from 1 to 7) as well as participants whose strength of intention was rather weak (i.e., 38.3% < 4.00), we were able to identify whether a minimum strength of intention was necessary for MCII to reduce meat intake more than information only control instructions. We observed that MCII participants who expressed a moderate or strong intention reduced their meat consumption more than participants in the control condition, whereas those who reported a weak intention did not. These results extend prior findings that demonstrated MCII effects for participants with moderate to strong intentions. In a study by Adriaanse et al. (2010a), for example, participants on average reported a strength of intention of 5.49 on a 7-point Likert scale with only 9.8% of the participants reporting an intention lower than 4.00. Together, these findings highlight the importance of moderate to strong intentions for MCII to support the attainment of desired outcomes.

### Limitations and Future Research

Several limitations of our study have to be noted, which lead us to suggestions for future research. First, the convenience sample from a student population limits the generalizability of the results. Participants’ baseline level of meat consumption was only about half the amount of the German average (BMELV, 2010). Hence, future research should aim at replicating our findings in more diverse samples.

Second, we had chosen a sample size in the range of prior similar studies (e.g., Adriaanse et al., 2010a). Still, it might be appropriate to extend the sample size of future studies in order to (a) be able to detect small effect sizes more reliably, and (b) to decrease the likelihood of baseline group differences despite randomization.

Third, although it is a strength of our study to at least include two follow-up measures, previous MCII studies provided evidence for the stability of effects on behavior change for up to 2 years (see Stadler et al., 2010). Accordingly, the inclusion of long-term follow-ups would be a valuable extension.

Fourth, we cannot unequivocally determine overall effect sizes of the MCII and control intervention *per se* on meat reduction, as no control group without any intervention was included. The lack of such a control group arose out of the primary focus to investigate differences between the classic information only intervention and the self-regulation focused MCII intervention. In future research, therefore, it would be worthwhile to include another control group receiving no intervention at all in order to determine and control for a possible meat reduction over time that is due to systematic study-unrelated variations in meat consumption (e.g., elicited by a food scandal that generated a lot of media attention). Still, the lack of such a control group does not affect our hypothesis and results on differences between the information + MCII group and the information-only control group.

Fifth, we suspect it was not only the provided information and MCII that contributed to changes in consumption but also asking participants to keep a diary, as doing so qualifies as a form of self-monitoring (see e.g., Zepeda and Deal, 2008). In support of this assumption, a review of health behavior change interventions identified self-monitoring as a particularly strong type of intervention (Michie et al., 2009). This is also consistent with our finding that participants in the information-only condition reduced their meat intake and with the feedback provided by some participants in our study, who stated that observing their own behavior this closely had been interesting as well as surprising as it revealed a higher consumption level than they would have expected. Thus, future research could vary the degree of self-monitoring to detect its distinct influence.

Sixth, the used diary measure could be improved. In general, diary approaches have the advantage to minimize biases compared to retrospective reports of consumption for a whole week (Schwarz, 1999). Whereas paper-based diaries are easiest to use for participants (Bolger et al., 2003), they bear the limitation of not tracking time of access. Future studies might thus provide insights into participants’ reporting behavior by using online food diaries (see for example Järvelä et al., 2006) or experience sampling technology (Bolger et al., 2003). Although participants might experience regularly accessing online food diaries or entering data on electronic devices as additional burden, such computer-based approaches seem a promising route for future research. Although compliance with regards to diary entries in the present study cannot be taken for granted, the high level of reported sincerity in participating, as well as the low amount of missing values and of diaries which were not returned, speak for a high quality of the data collected. It has also been pointed out that diary data are vulnerable to distortions through social desirability. As more objective measures in the context of assessing food intake, photographic diaries have been proposed (see for example Zepeda and Deal, 2008; Martin et al., 2009; Staiano et al., 2012) which could be a further valuable extension in future research. However, they increase the effort for participants and are costly. The finding that perceived demand from research staff did not differ between the MCII and control condition supports the assumption that social desirability does not explain the observed differences between conditions in the present study.

Seventh, our measure of meat consumption is limited by us providing weight categories in contrast to demanding exact weight information from participants. However, by giving examples and encouraging participants to consult weight information on packages, participants should have been able to report rather precisely, how much meat they had eaten.

Finally, we do not know how the participants who consumed less meat substituted their meat intake. Future research could thus extend the diary to other types of food.

### Implications

Food choices have been claimed to be among the most relevant areas for a transformation toward a more sustainable society, and animal products are named as specifically important “because more than half of the world’s crops are used to feed animals, not people. Land and water use, pollution with nitrogen and phosphorus, and (greenhouse gas) emissions from land use and fossil fuel use cause substantial environmental impacts” (UNEP, 2010, p. 80). For example with regard to climate change, Hedenus et al. (2014) calculated that, under current trends of the worldwide consumption of animal products, food-related greenhouse gas emissions may increase to a carbon dioxide (CO_2_)-equivalent emission level until 2070 which is likely to be larger than the total CO_2_-equivalent emission level compatible with meeting the hoped for 2°C limit of temperature rise. Westhoek et al. (2014, p. 196) report that halving the consumption of animal products in the European Union “would achieve a 40% reduction in nitrogen emissions, 25–40% reduction in greenhouse gas emissions and 23% per capita less use of cropland for food production.” Apart from that, these dietary changes are also expected to lower various health risks (Tilman and Clark, 2014; Westhoek et al., 2014). Hence, supporting individual intentions to attempt dietary changes of their meat consumption seems an important societal goal, specifically in light of the difficulties individuals report with acting on their intentions (Lea et al., 2006).

Our study shows that MCII can be applied as a strategy to support behavior change. MCII empowered individuals with moderate and strong intentions to reduce their meat consumption to translate their intention into behavior. Our research complements a growing body of evidence confirming that MCII is a useful intervention approach in helping people to attain their desired futures. Hence, we suppose that MCII should also be useful for facilitating other sustainable consumer behaviors (e.g., other food purchasing or eating behaviors such as choosing a regional/seasonal diet or organic products, but also mobility choices or energy use), which individuals intend to perform but perceive as difficult to implement.

## Conclusion

In line with previous findings, the present study suggests that people who engage in the self-regulation strategy of MCII change their behavior in the service of solving societal problems. Regarding the challenge to consume more sustainably, MCII helped individuals who were motivated to reduce their meat intake to realize an actual behavior change. We thus showed that MCII aligns behavior change to individuals’ respective intentions and narrows the intention-behavior gap. Future studies using MCII may aim at supporting sustainable behavior not only pertaining to meat intake but also to other pressing issues of responsible consumption.

## Author Contributions

LL was involved in formulating the research question, designing the study, collecting and analyzing the data, and writing the article. FW was involved in formulating the research question, designing the study, and writing the article. PG and GO were involved in interpretating the data, writing the article, and revising it critically for important intellectual content. All authors were responsible for drafting and approving the final manuscript. Lastly, all authors agree to be accountable for all aspects of the work in ensuring that questions related to the accuracy or integrity of any part of the work are appropriately investigated and resolved.

## Conflict of Interest Statement

The authors declare that the research was conducted in the absence of any commercial or financial relationships that could be construed as a potential conflict of interest. The reviewer LG and handling Editor declared their shared affiliation, and the handling Editor states that the process nevertheless met the standards of a fair and objective review.

## Footnotes

1 Accounting for the concerns raised by Simmons et al. (2011), we consider it relevant to disclose that our study additionally included a third intervention condition. This third condition did not address this article’s research question and did not affect our hypothesis and results. It was run for educational purposes. Information can be found in the Supplementary Material.

2 Further measures beyond the scope of this article’s research question are not explicated here. They did not affect our hypothesis and results. Information can be found in the Supplementary Material.

3 The decision not to restrict the text to environmental outcomes of meat consumption only was based on the recommendation by Tobler et al. (2011) that information interventions on sustainable food consumption should take both environmental and other arguments for sustainable consumption (e.g., health arguments) into account.
