# Supplementary material for: Supporting Sustainable Food Consumption: Mental Contrasting with Implementation Intentions (MCII) Aligns Intentions and Behavior
Source: Front Psychol. 2016 Apr 29;7:607. doi: 10.3389/fpsyg.2016.00607 (PMC4850472; doi:10.3389/fpsyg.2016.00607)
Supplement: Supplementary file 1 [file Table_1.DOCX]

Supplementary Material

Supporting Sustainable Food Consumption: Mental Contrasting with Implementation Intentions (MCII) Aligns Intentions and Behavior

Laura S. Loy*, Frank Wieber, Peter M. Gollwitzer, Gabriele Oettingen

*** Correspondence:** laura.loy@uni-hohenheim.de

In the following, we will give additional information on contents of the study, which are not covered in the main article (see Footnotes 1 and 2). Furthermore, we provide information on the MCII intervention’s wording.

# Information on the third experimental condition

As the study was run within a master thesis, a third condition was included for educational purposes. Thinking about ways to further support behavior change, we wondered whether social support might be helpful to increase the effectiveness of the MCII intervention. We included a collaborative MC + individual II condition in which participants discussed their future visions and obstacles with an interaction partner before planning goal implementation. Hence, in total, 120 individuals were randomly allocated to either the information-only control condition (*n* = 30), the information + individual MCII condition (*n* = 30), or the information + collaborative MC + individual II condition (*n* = 60). The data of participants in this third condition have been analyzed in the thesis of the first author and are not further explicated here.

# Information on additional measures

Other measures were included in the study, which do not address the research question we focus on in the main article. They did not affect our hypothesis and results, but we list them in the following in order to transparently provide a complete picture of the study’s contents (see Table 4). All questionnaires were computer-based. Participants answered Baseline-Questionnaire 1 during the first laboratory meeting and Baseline-Questionnaire 2 during the second laboratory meeting before the intervention. Questionnaire 3 was provided immediately after the intervention, still in the laboratory. We sent participants a link for Questionnaire 4 via email one week after the intervention (Follow-up 1) and a link for Questionnaire 5 four weeks after the intervention (Follow-up 2).

Table 4. Measures Applied in the Study

| Application | Questionnaire | Measures |
| --- | --- | --- |
| Laboratory Meeting 1 | Questionnaire 1 | Demography, Past behavior, Habit strength, *Need for cognitive closure, Need to belong* |
| Laboratory Meeting 2 | Questionnaire 2 | Intention, *Attitude, Perceived behavioral control, Norm, Commitment, Aspiration, Expectation, Incentive* |
|  | Questionnaire 3 | *Commitment, Expectation, Incentive, Shared reality (collaborative condition only)* |
| Link via Email | Questionnaire 4 | *Habit strength, Attitude* |
| Link via Email | Questionnaire 5 | Importance of reasons for reducing meat consumption, Study evaluation*, Habit strength, Attitude, Message evaluation, Sustainable consumption* |
| *Note.* Measures in italics are not reported in the main article, as they do not address its research question. | | |

# MCII Material

We gave the following instructions for the MC procedure (here translated from German into English). After each question, we provided ample writing space.

*Which goal would you like to set for yourself concerning meat consumption for the coming weeks? It should be challenging and specific. My goal: ……*

*What are positive aspects you associate with realizing your goal? You can think of the text you read. But also state everything else, which is important to you. ……*

*Now, please name the most positive aspect for you personally and write down a key word. The best aspect: ......*

*Imagine the events and experiences you associate with this aspect. What will change for you, if you realize your goal and this best aspect comes true? Let your mind go and write down your thoughts and imaginations. ……*

*Sometimes goals are not met, even if one has wished for it. Can you imagine something standing in the way of your goal? For everyone other obstacles are of main importance. What could be important obstacles for you personally? ……*

*If several obstacles came to your mind, please name the two most important ones and write down one or two keywords each.*

*Important obstacle 1: ...... Important obstacle 2: ......*

*Imagine the events and experiences you associate with these obstacles. Let your mind go and write down your thoughts and imaginations. Obstacle 1: ...... Obstacle 2: ......*

Instructions to phrase four implementation intentions, one of each type for each of two obstacles, read as follows:

*What can you do to overcome the obstacle? In which situation, when and where? Please formulate your own example following this structure: “If the situation (the obstacle) arises, then I will do something specific to overcome the obstacle.”*

*What can you do to prevent the obstacle? In which situation, when and where? Please formulate your own example following this structure: “If the situation (to prevent the obstacle) arises, then I will do something specific to prevent this obstacle.”*
